# Potential mechanisms of resistance to current anti-thrombotic strategies in Multiple Myeloma

**DOI:** 10.20517/cdr.2021.115

**Published:** 2022-03-07

**Authors:** Claire Comerford, Siobhan Glavey, Jamie M. O’Sullivan, John Quinn

**Affiliations:** ^1^Irish Centre for Vascular Biology, School of Pharmacy and Biomolecular Sciences, Royal College of Surgeons in Ireland, Dublin D02YNVV, Ireland.; ^2^Department of Haematology, Beaumont Hospital, Dublin D09V2N0, Ireland.; ^3^Hermitage Medical Clinic, Old Lucan Road, Dublin D20W722, Ireland.; ^4^Department of Pathology, Royal College of Surgeons in Ireland, Dublin D02YNVV, Ireland.; ^5^School of Medicine, Royal College of Surgeons in Ireland, Dublin D02YNVV, Ireland.

**Keywords:** Multiple myeloma, heparin resistance, thromboprophylaxis, venous thromboembolism, thrombin generation testing, endothelium, immunoglobulin

## Abstract

Multiple Myeloma (MM) is a common haematological malignancy that is associated with a high rate of venous thromboembolism (VTE) with almost 10% of patients suffering thrombosis during their disease course. Recent studies have shown that, despite current thromboprophylaxis strategies, VTE rates in MM remain disappointingly high. The pathophysiology behind this consistently high rate of VTE is likely multifactorial. A number of factors such as anti-thrombin deficiency or raised coagulation Factor VIII levels may confer resistance to heparin in these patients, however, the optimal method of clinically evaluating this is unclear at present, though some groups have attempted its characterisation with thrombin generation testing (TGT). In addition to testing for heparin resistance, TGT in patients with MM has shown markedly varied abnormalities in both endogenous thrombin potential and serum thrombomodulin levels. Apart from these thrombin-mediated processes, other mechanisms potentially contributing to thromboprophylaxis failure include activated protein C resistance, endothelial toxicity secondary to chemotherapy agents, tissue factor abnormalities and the effect of immunoglobulins/“M-proteins” on both the endothelium and on fibrin fibre polymerisation. It thus appears clear that there are a multitude of factors contributing to the prothrombotic milieu seen in MM and further work is necessitated to elucidate which factors may directly affect and inhibit response to anticoagulation and which factors are contributing in a broader fashion to the hypercoagulability phenotype observed in these patients so that effective thromboprophylaxis strategies can be employed.

## INTRODUCTION

### Background

Multiple Myeloma (MM) is a common haematological malignancy that remains a significant therapeutic challenge despite major advances in both treatments and biological understanding of the disease. Almost all patients will suffer relapse and in conjunction with this, MM is associated with a high rate of venous thromboembolism (VTE). It has long been acknowledged that there is a strong association between cancer and thrombosis but the risk of these events varies widely across the spectrum of cancer subgroups. VTE rates remain high in patients with MM with almost 10% of patients suffering from thrombosis during their disease course^[1,2]^.

**Figure 1:**
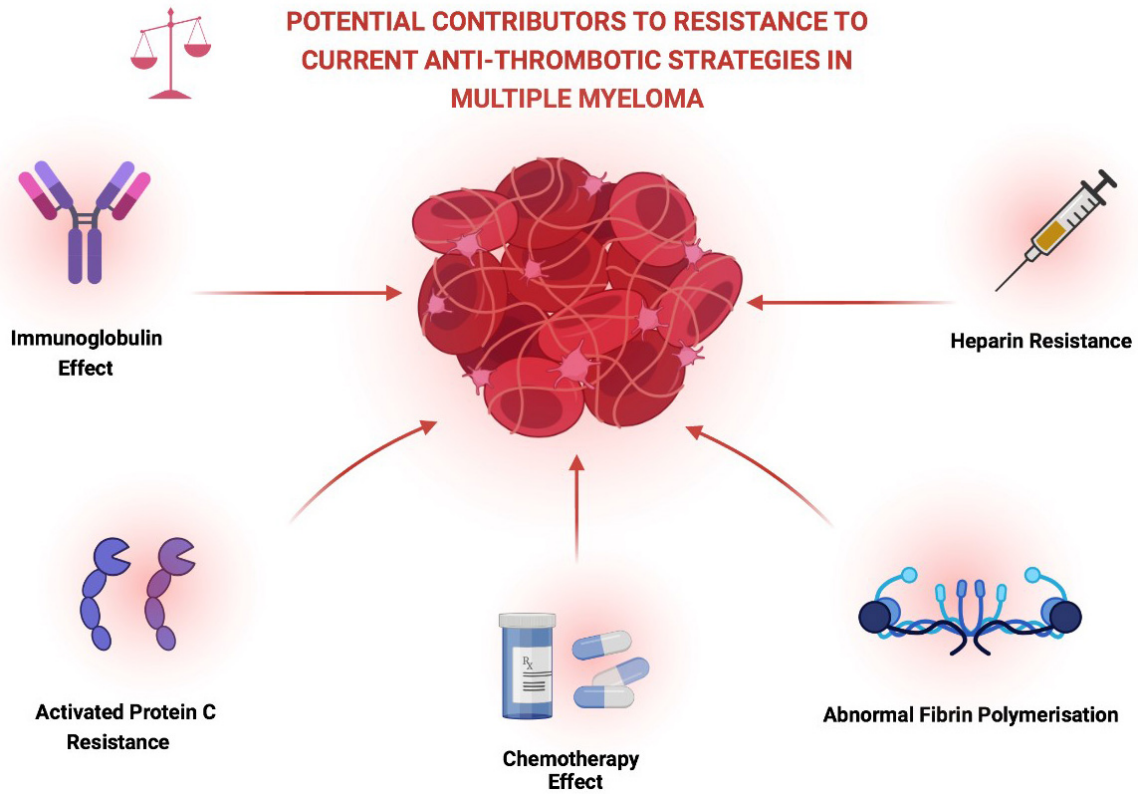
Description of some of the possible contributory factors to the persistently elevated VTE rates seen in multiple myeloma. Created with BioRender.com.

The occurrence of a thrombotic event in patients with MM can be associated with significant morbidity and can lead to interruption of treatment, requirement for anticoagulation use and indeed may preclude the use of certain agents in future treatment regimens. Certain studies have also suggested that patients with MM who suffer a thrombotic event are also more likely to have poorer overall survival though further confirmatory data is awaited in this area^[3,4]^. In light of these issues, optimisation of thromboprophylaxis and further understanding of the mechanisms conferring resistance to and failure of current anti-thrombotic strategies is an ongoing critical area of research [Figure 1].

### Treatment of MM

The treatment of MM has rapidly evolved over the past number of years with many therapeutic options now available. Triplet/three drug combinations are the initial standard of care for induction therapy though choice of treatment will depend on factors such as patient fitness, local treatment guidelines and availability/funding of therapy^[5,6]^. Generally, in younger, fitter patients who may be eligible for autologous transplantation (ASCT), initial therapy will comprise of a proteasome inhibitor such as bortezomib and either an immunomodulatory (IMiD) agent such as lenalidomide or an alkylating agent such as cyclophosphamide. These drugs are usually combined with dexamethasone, a corticosteroid, for maximum efficacy^[5]^. There are also very efficacious treatments available for older patients or those who may not be fit for ASCT including a combination of lenalidomide and dexamethasone or VMP therapy (bortezomib, melphalan and prednisolone)^[5,7]^. Upon relapse of disease, treatment options include carfilzomib (a second generation PI), alternative IMiDs such as pomalidomide and anti-CD38 monoclonal antibodies including daratumumab and isatuximab^[5,7]^. It is well recognised that IMiD therapy confers a high thrombotic risk to the patient, particularly when combined with high doses of steroids, but the exact thrombotic risks of many other agents have been less well characterised^[8,9]^.

### Risk assessment of venous thromboembolism

Patients with MM have a multitude of risk factors for development of thrombosis. These can generally be categorised into patient related factors such as prior VTE and increased body mass index (BMI), MM related factors (e.g., renal impairment, hyperviscosity, disease activity, reduced mobility) and therapy-related risk factors. There are a number of models and predictive scores that have attempted to stratify individual risk for VTE. The Khorana score is widely used to identify risk for cancer-associated VTE in patients with malignancy who are initiating systemic therapy. It is comprised of five clinical and laboratory indices, including type of cancer, platelet count, haemoglobin level and white cell count (WCC) as well as BMI^[10]^. However, given that it is largely based on solid organ tumour data, it has been recognised that this score likely has limitations in the MM population with several studies observing that it does not accurately predict VTE risk within this cohort^[2,11,12]^. In fact, in a study of over 300 patients with MM, Barrett *et al.*^[2] ^noted that WCC was the only single variable from the Khorana score that retained predictive significance for VTE in their cohort.

The current International Myeloma Working Group (IMWG) has provided guidance, based on stratification by Palumbo *et al.*^[13]^, on thromboprophylaxis in MM by compiling a VTE risk assessment incorporating individual, therapy- and myeloma-related factors. This guidance has also been incorporated into the National Comprehensive Cancer Network (NCCN) guidelines but several groups have observed that these guidelines do not fully capture VTE risk and thus have sought to develop novel and more rigorous VTE risk assessment tools [Table 1]. Sanfilippo *et al.*^[12]^ explored individual risk factors for VTE and combined the relevant factors to develop a risk prediction model called the “IMPEDE VTE” score which attributes scores to each contributory risk factor. Similarly, Li *et al.*^[14] ^evaluated the performance of the current NCCN guidelines in a large population based cohort while simultaneously developing their own VTE risk-assessment model specifically for patients undergoing therapy with IMiDs. They concluded that their own model, the SAVED score, which incorporated recent surgery, Asian ethnicity, prior history of VTE, age > 80 and dexamethasone dose had a greater discriminative power than the current consensus recommended by the NCCN guidelines^[14]^. While these scoring algorithms have advanced VTE-risk discrimination in MM, some concerns have been raised that these scores were designed using retrospective data which was based upon therapies that are now less commonly used^[15]^. Incorporation of additional biomarkers may further improve the accurate risk prediction of VTE for this vulnerable cohort.

**Table 1:** Recently developed risk stratification tools for VTE in multiple myeloma

**IMPEDE VTE score** **Variable**	**Score**	**SAVED score** **Variable**	**Score**
**I**MiD (immunomodulatory agent)	4	Recent **s**urgery (within last 90 days)	2
Body **m**ass index (>/ 25 kg/m^2^)	1	Ethnicity: **A**sian race	-3
Fracture (**p**elvic, hip or femur)	4	Prior history of **V**TE	3
Use of **e**rythropoietin-stimulating agent	1	Age “**e**ight” > 80	1
Use of **d**oxorubicin chemotherapy	3	**D**examethasone use (standard or high)	1 or 2
Use of **d**examethasone (high-dose/low-dose)	4/2	**Low risk: </ 1** **High risk: >/ 2**	
**E**thnicity = Pacific Islander/Asian	-3		
Prior **V**TE (preceding MM)	5		
Presence of **t**unneled line	2		
Current/**e**xisting use of therapeutic LMWH/warfarin	-4		
Current/**e**xisting use of prophylactic LMWH/aspirin	-3		
**Low risk: </ 3** **High risk: 4-7** **Very high risk: >/ 8**			

Adapted from Ref.^[12,14]^. VTE: Venous thromboembolism; MM: Multiple Myeloma.

### Current anti-thrombotic regimens in MM

It is well recognised that due to the unacceptably high thrombosis rates in MM, pharmacological thromboprophylaxis is often necessitated to try mitigate this risk. The IMWG recommend the use of aspirin in lower risk patients and either prophylactic-dose low molecular weight heparin or treatment-dose warfarin in patients at higher risk^[13]^. In more recent times, focus has switched to evaluating the effectiveness of the direct oral anticoagulant medications (DOACs) including the factor Xa inhibitors (apixaban, rivaroxaban) and factor II inhibitors (dabigatran) in prevention of VTE in MM. While DOACs have been shown in several large trials to be effective and safe in both treating and preventing VTE in patients with malignancy, very few studies have sought to confirm this effect specifically in cohorts of MM patients^[16-18]^. Despite this, a number of small scale studies have cautiously reported their experiences of the safe and effective use of DOACs in MM.

Storrar *et al.*^[19] ^have described their experience of apixaban thromboprophylaxis in 70 patients with MM, all receiving IMiDs as part of induction therapy. These patients were treated with apixaban 2.5 mg twice daily and there were no reports of VTE during the first six months of therapy with low rates of major bleeding also observed. They concluded that apixaban appeared to be a safe and attractive option for this cohort of patients. Similarly, Cornell *et al.*^[20] ^reported their experiences of apixaban thromboprophylaxis in a more heterogenous group of 50 patients with MM undergoing IMiD-based therapy at various timepoints of the disease course. Again, no instances of VTE were observed in the first six months of observation and, reassuringly there were also no instances of major haemorrhage during this time period. They too concluded that low-dose apixaban was safe and well-tolerated as primary prevention of VTE in patients receiving IMiD therapy. These are, however, small-scale studies and larger, prospective studies will be required to provide conclusive evidence for the long term use of DOACs in MM.

### VTE rates despite thromboprophylaxis: immunomodulatory agents

The rate of VTE occurrence remains unacceptably high in MM patients with approximately 10% of patients developing a thrombosis during the course of their disease^[21,22]^. It has been demonstrated that thrombosis rates in patients with malignancy are highest in the first year following diagnosis and in particular, in MM, thrombosis rates appear to be highest in the first 3 months following diagnosis^[1,13,22,23]^. It is hypothesised that these initial high rates are due to both accelerated neoplastic activity, increased tumour burden and, with treatment initiation, there may be overwhelming release of procoagulant factors and cytokines once apoptosis ensues. Given the particularly high risk of VTE associated with IMiDs, adjuvant pharmacological thromboprophylaxis is now widely in use. However, despite this, many groups have reported that VTE rates remain persistently elevated. Leclerc *et al.*^[22] ^reported their observations from a cohort of 213 patients who had received IMiD therapy where thrombotic events were reported in 18.3% of patients with more than half of these events occurring during the first three months of therapy, during which time the vast majority of patients were receiving thromboprophylaxis. VTE occurred more often with anti-platelet use rather than with anticoagulation, an observation that was also seen in the MELISSE study^[22,24]^.

Bradbury *et al.*^[25] ^also recently described the thrombosis rates during the Myeloma IX and Myeloma XI trials. Both trials are phase 3 trials incorporating the upfront use of an IMiD and corticosteroid-containing regimens as induction therapy with the Myeloma IX study containing data from 1936 patients and the Myeloma XI study containing data from 4358 patients. Due to the interim development of the aforementioned IMWG risk assessment guidelines, there were higher rates of thromboprophylaxis (80.5% *vs.* 22.3%) in the later Myeloma XI study but despite this, there was only a modest reduction in thrombosis rates in the latter trial. In the Myeloma IX trial, the 6 months cumulative thrombosis incidence rate was 20.7% in the CVAD (cyclophosphamide, vincristine, adriamycin, dexamethasone) group (non IMiD based) and 15.0% in the CTD (cyclophosphamide, thalidomide, dexamethasone) group (IMiD containing) while in the Myeloma XI trial, 6 months cumulative incidence of VTE was 10.7% in the CRD (cyclophosphamide, lenalidomide, dexamethasone) group and 11.7% in the CTD group, thus showing that despite adherence to current thromboprophylaxis guidelines, VTE rates remain disappointingly high^[25]^.

### High VTE rates with other anti-myeloma therapies

Despite IMiDs often being regarded as the culpable prothrombotic agent, consideration has to be given to the thrombotic risk posed by other classes of drugs used to treat MM and worryingly, some of the more novel agents appear to be increasingly linked with thrombosis. Carfilzomib is a second generation proteasome inhibitor with reported VTE rates of 5%-14% in clinical trials and although thromboprophylaxis is recommended for MM patients receiving carfilzomib-containing regimens, the most effective thromboprophylaxis strategy remains to be determined, nor has the mechanism underlying carfilzomib-associated thrombosis been adequately studied^[26-29]^. The findings of Piedra *et al.*^[29]^ suggest that these patients might benefit from a more enhanced thromboprophylaxis regimen. They recently reported their observations from a retrospective study of VTE incidence across different induction regimens used in newly diagnosed MM (NDMM). They compared rates between KRD (carfilzomib, lenalidomide, dexamethasone) + aspirin, RVD (lenalidomide, bortezomib, dexamethasone) + aspirin and KRD + rivaroxaban and found significantly differing rates of VTE depending on thromboprophylaxis agent used with rates of 16.1%, 4.8% and 4.8% observed in the groups respectively. This suggests that carfilzomib may be more thrombogenic than bortezomib (when combined with lenalidomide), a first generation proteasome inhibitor, though this risk appeared to be somewhat mitigated by using a DOAC rather than an anti-platelet agent^[29]^. In addition to this, recent advances in cellular therapy have led to the availability of anti-BCMA chimeric antigen receptor T (CAR-T) cell therapy in MM. CAR T-cell therapy is associated with high rates of VTE with Parks *et al.*^[30]^ reporting almost 1 in 10 patients with either non-Hodgkin’s lymphoma or MM reportedly developing VTE in the first 60 days post CAR-T cell infusion. There is currently a poor understanding of the pathogenesis of same or indeed on the optimal preventative and therapeutic strategies^[30]^.

## POSSIBLE MECHANISMS OF RESISTANCE: THROMBIN-MEDIATED

### Heparin resistance

#### Potential mechanisms contributing to heparin resistance

As discussed above, many patients with MM who are deemed at high risk of VTE are administered thromboprophylaxis with LMWH and it is also commonly utilised at therapeutic doses for treatment of thrombotic episodes in MM. Heparin binds to antithrombin and accelerates the interaction between antithrombin and thrombin or antithrombin and factor Xa, both of which should result in inhibition of prothrombotic activity. Heparin resistance is often defined as the need for higher heparin doses to achieve a targeted level of anticoagulation but it may be multifactorial and its identification is complicated by the lack of consensus on the appropriate target levels in conjunction with the optimal method for measuring heparin effect^[31]^.

One of the important mechanisms responsible for heparin resistance is deficiency of antithrombin (formerly known as antithrombin III). Many disease states such as liver failure, sepsis and treatments, such as asparaginase use in acute leukaemia, are associated with reductions in antithrombin levels but the prevalence of antithrombin deficiency in MM is far less well known and has not been evaluated on a large scale basis^[31,32]^. It is however, well described that significant proteinuria can contribute to thrombotic potential and that reduced serum albumin levels are associated with increased urinary protein loss, including that of antithrombin^[33]^. This may be of particular significance both in patients with MM and amyloidosis given the high rates of kidney impairment seen in these conditions. Thus, certain groups have highlighted the need for consideration for testing for antithrombin deficiency in patients with MM in the context of thrombosis and significant proteinuria^[21]^.

Aside from antithrombin deficiency, an additional factor that may contribute to heparin resistance is presence of high levels of coagulation Factor VII (FVIII). Increased doses of heparin have been necessitated for effective thromboprophylaxis in patients who exhibit high levels of FVIII such as patients suffering with acute infective/inflammatory conditions such as Covid-19^[31]^. Increased FVIII levels in MM have been reported by several groups with particular elevations seen in those undergoing IMiD therapy^[34-36]^. This raises the question that perhaps higher doses of heparin are necessitated in such patients to achieve adequate protective anticoagulation levels and thus reduce the high rates of VTE seen with current standard dosage.

#### Evaluating heparin resistance with thrombin generation testing

Thrombin generation assays (TGAs) are global haemostasis assays that seek to assess the entire coagulation process and are thought to be more sensitive than a standard coagulation profile given that the endpoint is conversion of fibrinogen to fibrin. They are *in vitro* assays that utilise tissue factor (TF) and phospholipids to activate the coagulation cascade. The concentration of thrombin is thus measured over a defined time period and a curve is generated to illustrate this production. Various measurements can then be extrapolated from the curve to describe thrombin activity such as lag time (time until thrombin initiation), peak thrombin generation and endogenous thrombin potential (ETP) which reflects the thrombin formation capacity of the plasma^[37]^. Several groups have postulated that patients with MM may be “resistant” to treatment with heparin and have sought to evaluate this resistance using TGAs.

Chalayer *et al*.^[38]^ describe their observations where they sought to characterise the effect of heparin thromboprophylaxis on thrombin generation in a cohort of patient with MM with a view to exploring potential mechanisms of heparin resistance. They compared a group of patients with MM with intermediate/high VTE risk factors with a group of patients hospitalised with respiratory illness who were deemed to have a low thrombotic risk. They observed similar ETP levels in both of the groups and concluded that there was no *in vitro *evidence of specific resistance to LMWH in this particular cohort of MM patients^[38]^. Gracheva *et al.*^[39]^ also assessed a variety of global haemostasis assays (including TGAs, thromboelastography and thrombodynamics) in a cohort of 59 patients, 25 with NDMM and 34 patients undergoing stem cell mobilisation. They compared their results with a cohort of healthy individuals and found that that both thrombodynamics and thromboelastography showed hypercoagulability in both sets of MM patients when compared with the healthy cohort. They then assessed TGAs in the patients undergoing stem cell mobilisation who were receiving heparin therapy and found widely varied results for each individual with no demonstration of hypocoagulability in some patients and more worryingly, a shift towards hypercoagulability in over 20% of patients. The authors concluded that the use of heparin therapy, at its current dose, was ineffective in these patients and that it may either indicate inadequate dosage or heparin resistance in this cohort^[39]^. Given the stark discrepancy between these studies, further research in larger, more heterogenous groups of MM patients would be necessitated to evaluate if heparin resistance is prevalent and clinically relevant in MM.

### Predicting VTE risk with thrombin generation assay testing

Thrombin generation has been studied extensively in malignancy and a large prospective observational study, the Vienna Cancer and Thrombosis Study, assessed the predictive value of thrombin generation for VTE in over 1000 patients with a variety of malignancies. They concluded that patients with an elevated peak thrombin (>/ 611 nM) had an increased risk for development of VTE with a hazard ratio of 2.1^[37]^. However, the numbers of MM patients included in this study were small thus several groups have sought to establish the predictive value of TGAs specifically in MM.

Leiba *et al*.^[40]^ tested thrombin generation in a cohort of 36 patients with MM at various timepoints over a 2.5-year period with the main objective of determining if thrombin generation has a predictive value for VTE. They observed significantly higher ETPs and peak height values in those who developed thrombotic events compare with those who did not. Interestingly, they also noted a gradual increase in thrombin generation parameters in the time period preceding the thrombotic event. Unlike the findings seen by Gracheva *et al.*^[39]^ they noted that anticoagulation therapy was associated with a significant decrease in ETP and peak height values. As previously discussed, there is an excess incidence of VTE in MM in the first year following diagnosis and the authors observed a moderate in increase in TG parameters concomitant with commencement of MM treatment suggesting that this may partially explain the timing of this excess incidence^[40]^. Overall, they concluded that thrombin generation testing, both at baseline and during therapy, could serve as a predictive tool for thromboembolic events and perhaps may also have a role in monitoring individual response to heparin^[40]^.

Fotiou *et al.*^[41]^ subsequently carried out a larger prospective study of patients with NDMM and sought to explore the hypercoagulability characteristics in the period of treatment naivety pre-induction therapy. They recruited 144 patients with NDMM and compared their results to a group of healthy individuals. One of their outcomes was measurement of thrombin generation and, in direct contrast to the above findings, they found that, overall, thrombin generation was attenuated in the MM patients compared to the healthy individuals with longer lag-times and lower peak values observed. They also found significantly reduced ETPs in the MM cohort indicating an overall reduced enzymatic activity of thrombin. Interestingly, certain lower ETPs appeared to correlate with a higher risk of VTE and the authors concluded that they had identified clinically relevant biomarkers for VTE risk stratification with their hypothesis being that attenuation of thrombin generation should be interpreted as a reflection of endothelial cell activation i.e., “exhausted thrombin generation”^[41]^. Legendre *et al.*^[42]^ also showed a potentially hypocoagulable state, with reduced peak thrombin and ETP values in a small group of 14 patients who were both chemotherapy and anticoagulant naïve.

Thus, there appears to be a marked discrepancy in the evaluation of thrombin generation in MM with both hyper- and hypo-coagulable states being observed by different groups, depending on timing of the testing and exposure of the patient to chemotherapy agents. Thrombin generation also increases with age and thus matching against younger, healthy control subjects may confound results^[42]^. Further evaluation in larger studies with stringent age-matching is necessary to determine both the possible predictive value of thrombin generation for VTE and the clinical utility of such testing in monitoring response and resistance to anticoagulant use (see Table 2 for result of thrombin generation testing).

**Table 2:** Results of thrombin generation evaluation in patients with MM

**Author(s)**	**Multiple Myeloma patient cohort evaluated**	**Evaluated heparin resistance**	**Evaluated predictive potential for VTE**	**Findings**
Chalayer *et al.*^[38]^ 2019	Patients on first line therapy with high VTE risk (*n* = 6)	Yes	No	No evidence for an in vitro LMWH resistance in those with MM compared to patients without MM
Gracheva *et al.*^[39]^ 2015	Patients with primary MM (*n* = 25) and patients with MM in remission (*n* = 34) undergoing blood stem cell mobilization	Yes	No	Possible “heparin resistance” with no heparin effect seen in 22% of patients and hypercoagulability seen in certain patients
Leiba *et al.*^[40]^ 2017	Patients with newly diagnosed disease (*n* = 13) and patients receiving therapy upon relapse (*n* = 23)	No	Yes	Patients who had a thrombotic event exhibited significantly higher ETP and peak height values than those who did not have a thrombotic event
Fotiou *et al.*^[41]^ 2018	Patients with newly diagnosed disease (*n* = 144)	No	Yes	Lower ETP values were associated with VTE occurrence
Legendre *et al.*^[42]^ 2017	Patients with newly diagnosed disease (before treatment, including anti-coagulation) (*n* = 14)	No	Yes	Hypocoagulable profiles including decreased ETP values

VTE: Venous thromboembolism; MM: Multiple Myeloma; ETP: endogenous thrombin potential.

### The potential role of thrombomodulin in pathogenesis of thrombosis

Thrombomodulin is a transmembrane glycoprotein which is located on the luminal surface of endothelial cells. It has roles in both regulation of coagulation and inflammation with its main function being to bind to thrombin. Binding of thrombin to thrombomodulin results in loss of thrombin’s procoagulant and profibrogenic properties and acquisition of the ability to activate protein C. It has thus been hypothesised that a reduction in thrombomodulin levels in MM may confer a hypercoagulable profile in patients.

Corso *et al.*^[43] ^sought to serially evaluate the changes in coagulation profiles in patients with RRMM both before and during/after treatment with thalidomide. While many markers were not found to significantly vary, they did observe significant variations in thrombomodulin levels. Thrombomodulin levels appeared to be slightly reduced in most patients at baseline but underwent further reductions during initial treatment with thalidomide therapy with the authors thus concluding that reductions in thrombomodulin levels may have a pathogenic role in thalidomide-related thrombosis. Fotiou *et al.*^[41]^ also noted a reduction in thrombomodulin levels in their cohort of 144 patients with NDMM. Zappasodi *et al.*^[44] ^subsequently sought to confirm the effect of thalidomide on thrombomodulin levels and evaluated the serial behaviour of thrombomodulin in 26 patients with RRMM who commenced thalidomide and dexamethasone therapy. Unlike their initial study where serum thrombomodulin levels fell during the first month of thalidomide therapy, they did not observe any significant modifications of thrombomodulin levels from baseline during the early stages of therapy. They did however note that the cohort of patients had low median basal values of thrombomodulin and thus concluded that this could perhaps suggest a thrombophilic state intrinsic to the MM disease process rather than then anti-myeloma therapy^[44]^. Again, these observations are limited not only by low patient numbers but also by lack of evaluation in patients being treated with the newer therapeutic agents that are currently in widespread use.

## OTHER POSSIBLE MECHANISMS OF RESISTANCE

### APC resistance

Many groups have sought to unravel the prothrombotic phenotype in patients with MM with common observations including significant elevations in von Willebrand Factor (VWF) antigen, Factor VIII, D-dimer and fibrinogen levels in patients with active disease. However, the contributory effect of these abnormalities towards VTE occurrence has not been fully disentangled thus far^[15,36,45-49]^. Interestingly, abnormalities of activated protein C (APC) appear to be a relatively common phenomenon exhibited in patients with MM and, unlike the above, do appear to significantly contribute to VTE occurrence. The normal biological role of APC is to inactivate Factor Va and Factor VIIIa with APC resistance (APCR) leading to non-cleavage of these factors and resultant increased thrombin generation which can potentially lead to a hypercoagulable state. In the Caucasian population, resistance to APC is one of the most common coagulation abnormalities associated with VTE and generally correlates with the presence of the Factor V Leiden R506Q mutation^[50]^. However, acquired APCR has been described in association with certain malignancies including MM^[51,52]^.

Given this association, Elice *et al.*^[53]^ sought to fully characterise the incidence of acquired APCR and its correlation with VTE in a large cohort of patients with MM (*n *= 1178). They recorded APCR in 109 patients (9%) but interestingly, this did not necessarily translate into the presence of factor V Leiden mutations with over half of these patients testing negatively for this mutation. Those with an abnormal APCR ratio but negative DNA testing for the factor V Leiden mutation were considered to have acquired APCR. Within this cohort of patients with acquired APCR, a higher incidence of VTE was observed when compared with controls (31% *vs.* 12%) and furthermore, they also exhibited a lower thrombosis-free survival. The authors thus concluded that the presence of acquired APCR was statistically associated with an increased VTE risk^[51,53]^.

Following on from this, other groups have sought to further unravel the mechanisms behind APCR and isolate the exact causative single nucleotide polymorphism (SNPs) in the endothelial protein C receptor (EPCR). EPCR has a key regulatory role in protein C activity via binding and facilitating the interaction with the thrombin-thrombomodulin complex. Several polymorphisms have been reported in EPCR with the 4678G/C SNP thought to be associated with high levels of circulating APC and reduced risk of thrombosis. Dri *et al.*^[54]^ sought to evaluate the presence of this polymorphism in a cohort of patients with MM who had developed thrombosis and found a significantly lower frequency of the 4678C allele of the EPCR gene in MM patients compared with the known frequency in a healthy adult population. The authors concluded that the reduced frequency of this protective allele could be contributing to overall disease hypercoagulability and that it may be useful to test for same to risk stratify MM patients. However, this was a small study and thus further evaluation of larger series of patients would be necessitated to determine that there is in fact an increased prevalence of this SNP in the MM population and if it indeed has a contributory effect to overall rates of VTE^[54]^.

### Endothelial dysfunction and toxicity related to the effect of chemotherapy 

With the widespread introduction of several novel therapies for MM, there has been a focus on emergent cardiovascular complications in conjunction with the persistence of high rates of VTE in the MM population^[26,55]^. Many of the hypotheses surrounding this VTE risk centre on the toxicity that may be caused to the endothelium by certain anti-myeloma agents. Dexamethasone is a corticosteroid that is used in many of the treatment regimens for MM. Studies have shown that combining dexamethasone with other agents increases a patients risk of VTE^[8,9]^. The reasons behind this are not well understood but there is some *in vitro* evidence that dexamethasone can stimulate the endothelium to increase expression of both VWF and TF^[56]^. Similarly, IMiD agents and carfilzomib are both thought to activate and cause direct toxicity to the endothelium with a downstream pro-thrombotic effect occurring following release of coagulation factors and cytokines^[57,58]^.

A small number of studies have been carried out specifically evaluating the toxic effects of anti-myeloma chemotherapy agents on endothelial cells (ECs). Early *in vitro *studies of IMiD agents noted that in a previously compromised/injured endothelium, as which would be seen with prior chemotherapy use, thalidomide could alter the expression of thrombin receptor PAR-1 and thus induce endothelial dysfunction and potentially a hypercoagulable state^[59]^. In a more recent study, Sanchez *et al.*^[60]^ have reported their observations on exposure of ECs to some of the newer MM induction chemotherapy agents and they have shown that these agents can in fact lead to increased VWF and ICAM-1 expression and also an increase in cell permeability, exhibited by reduced VE-cadherin expression and cell monolayer integrity. Interestingly, this effect appeared to be somewhat counteracted by addition of the endothelial protectant agent defibrotide^[60]^, thus raising the hypothesis that perhaps endothelial protection agents could have some anti-thrombotic utility in MM.

### The direct effects of M-protein/immunoglobulin on the prothrombotic environment and fibrin clot formation

MM is characterised by high levels of circulating clonal immunoglobulins or “M-proteins” and these immunoglobulins are hypothesised to have the ability to contribute directly to thrombotic risk, not only by direct vascular wall injury but also by interference with fibrinolysis^[61]^. Fibrinolysis is the breakdown of fibrin within blood clots and is a highly regulated enzymatic process that, if functioning correctly, prevents unnecessary accumulation of intravascular fibrin. Clot stability is dependent upon many factors including local factors such as calcium concentration, thrombin concentration, pH and platelets numbers^[62]^. It is also influenced by the geometric compilation of the fibrin network and the composition and diameter of the fibrin fibres from which it is constructed^[62]^. Abnormal fibrin clot structure can lead to prolonged resolution/retraction of a clot^[63]^.

Carr *et al.*^[64]^ originally sought to evaluate the influence of high levels of immunoglobulin on fibrin polymerisation and clot structure *in vitro*, noting that in congenital dysfibrinogenemia, recurrent thrombosis were associated with the presence of abnormally thin fibres of fibrin. They hypothesised that, given that fibrin structures are sensitive to the environment in which they are formed, that high levels of immunoglobulin would interfere with fibrin monomer polymerisation. They characterised the clot formation in MM and , as seen in congenital dysfibrinogenemia, they noted that the clots were associated with the presence of abnormally thin fibrin fibres^[64]^. They subsequently sought to evaluate the resistance of such clots to fibrinolysis and observed that these clots dissolved at a slower rate and they thus concluded that they were associated with inhibition of fibrinolysis^[65]^.

Undas *et al.*^[66] ^subsequently sought to evaluate fibrin clot properties and their determinants *ex vivo* in a cohort of 106 MM patients. Following collection of peripheral blood from these patients, they performed fibrin clot analysis, measuring variables such as clot permeation, clot compaction, turbidity measurements and also the efficiency of fibrinolysis using plasma clot lysis assays. They observed that plasmin clot variables differed significantly between the MM patients and a cohort of healthy control subjects with changes including higher fibrin fibre densities and reduced tPA-mediated lysability. They hypothesised that higher thrombin generation potentials were likely a major mechanism in these alterations in clot properties and that this abnormal fibrin clot structure likely exhibited a prothrombotic phenotype^[66]^.

The concept that local thrombin concentrations can impact upon the structure of clots with higher thrombin concentrations generating more stable clots which are more resistant to fibrinolysis and may promote thrombosis has been well described^[62,63,67,68]^. It thus appears possible that in MM, given the duel pathology of both high circulating immunoglobulin levels and potentially high thrombin generation, clots produced could be less susceptible to fibrinolysis and thus perhaps more resistant to the current anticoagulant strategies that are employed.

### Tissue factor

TF is an essential glycoprotein that serves as principal initiator of coagulation. TF-mediated conversion of Factor IX to its activated form is pivotal for effective haemostasis while disruption of the endothelium causes exposure of TF-expressing endothelial cells and thus facilitates binding of factor VII^[69]^. Apart from its roles in coagulation, TF has also been implicated in both tumour spread and angiogenesis and in conjunction with this, high levels of TF have also been described in several solid organ malignancies with conflicting reports on its potential utility as a biomarker for VTE occurrence^[70]^. TF can also be demonstrated on circulating microparticles (MPs) which are small, procoagulant membrane vesicles that are released from cells during activation or during apoptosis. These MPs are found in low steady state concentrations in healthy individuals and increase during times of inflammation. MPs carrying TF express platelet selectin glycoprotein ligand-1 which binds p-selectin on the surface of activated endothelial cells^[70]^.

In light of the above, several groups have sought to establish the role of TF in MM disease. Papageorgiou *et al.*^[71]^ investigated the role of TF and MP-TF *in vivo *in human myeloma cell lines. They determined that the expression of the TF gene, the presence of the TF protein on cell membrane and the procoagulant activity of TF were all detectable. They also observed that these cells could release MPs into their microenvironment and that these MPs conferred markedly increased procoagulant activity, expressing twofold higher levels of TF as compared to the original cells. The authors thus concluded that the hypercoagulability observed in MM may be enhanced by myeloma cell derived MPs released into the microenvironment^[71]^.

Other groups sought to evaluate the TF activity in patients with MM with Auwerda *et al.*^[72] ^evaluating this in a cohort of 122 patients with MM. They found that MP-TF activity levels were increased in the MM cohort in comparison to healthy volunteers. They performed serial evaluation of these activity levels and found that MP-TF activity levels showed a reduction post-induction therapy in many of the patients, but interestingly, levels remained elevated in those who had suffered a thrombotic episode^[72]^. Nielsen *et al.*^[73]^ also sought to explore the role of TF in MM by isolating extracellular vesicles (EVs) from the peripheral blood of 20 patients with MM and subsequently demonstrating substantially higher thrombin generation and TF activity when compared with healthy control subjects. Similar to the above, they also performed serial activity levels and noted that the procoagulant activity of the EVs diminished after treatment, though of note, neither of the treatment groups that they evaluated contained the more thrombogenic IMiD agents (treatment groups = bortezomib, cyclophosphamide and dexamethasone and melphalan, prednisolone and bortezomib)^[73]^.

All groups concluded that their findings may, at least in part, explain why there is an increased risk of VTE in MM but the prothrombotic contribution of TF in MM would need to be confirmed in larger cohorts of patients. In addition to this, TF activity is not routinely measured in clinical practice and a standardised assay would have to be developed and validated before it could be considered for widespread use as a biomarker. 

## CONCLUSION

It is clear that rates of thrombosis remain high in MM and, unfortunately, current thromboprophylaxis regimens do not entirely abrogate this risk of VTE. Thus the gap in knowledge between the persistently high levels of VTE and the inadequacies in current preventative measures needs to be bridged.

It is also increasingly evident that there are a multitude of factors contributing to the prothrombotic milieu seen in MM, many of which cannot be evaluated in a clinical setting. Further work is necessitated to elucidate specific pathways and factors contributing to the hypercoagulable phenotype observed in these patients. Additional clinical studies should focus on identifying novel biomarkers of VTE risk in MM and developing effective screening methods for anticoagulant resistance. Taken together, these approaches may help inform therapeutic strategies to overcome these mechanisms of resistance and provide effective thromboprophylaxis for patients with MM.

## DECLARATIONS

### Copyright

© The Author(s) 2022.
